# Prolonged Use of Portable Listening Devices Among Medical Students in a Healthcare Institution in Puducherry: A Clinico-Audiological Study

**DOI:** 10.7759/cureus.78929

**Published:** 2025-02-13

**Authors:** Catherine Floria, Prabu Velayutham, Prem Davis

**Affiliations:** 1 Otorhinolaryngology - Head and Neck Surgery, Sri Venkateshwaraa Medical College Hospital and Research Centre, Puducherry, IND; 2 Otorhinolaryngology - Head and Neck Surgery, Central University of Tamil Nadu, Thiruvarur, IND

**Keywords:** audiometry, hearing loss, mobile, noise, students

## Abstract

Introduction

Mobile phones are commonly used in daily life. Nowadays, many young adults listen to portable listening devices at high volumes for extended periods, resulting in prolonged exposure to loud noise. This can lead to hearing loss, which not only impacts the patient’s quality of life but also interferes with their daily activities.

Aim

The aim of this study is to assess the clinical impact of portable listening devices by examining clinical symptoms and findings among medical students who use these devices and evaluating their hearing thresholds.

Methods

This cross-sectional study was conducted among medical students at Sri Venkateshwaraa Medical College Hospital and Research Centre in Puducherry, India, between January 2020 and April 2020. Following the initial assessment, all participants underwent pure-tone audiometry (PTA) in a soundproof room to measure their hearing thresholds, and the results were analyzed.

Results

A total of 236 medical students participated in this study, with a mean age of 21.1 ± 0.86 years. Among them, 142 (60%) were female. The most common symptom reported was ear pain, experienced by 33 participants (14%). PTA (bone conduction) results showed that 227 (96%) participants had thresholds of ≥5 dB in the right ear, while 223 (94%) had the same in the left ear. Additionally, 139 (59%) of participants had hearing thresholds (air conduction) between 15 and 20 dB in the right ear, and 97 (41%) in the left ear. The study found a significant association between gender (p = 0.015) and hours of daily device usage (p < 0.01).

Conclusions

These results suggest that prolonged exposure to portable listening devices increases the risk of hearing impairment. To prevent gradually progressive noise-induced hearing loss, proactive measures must be taken to reduce extended exposure to these devices, safeguarding both the current younger generation and future generations.

## Introduction

Mobile phones have become an integral part of daily life, with their usage increasing exponentially due to the diverse functionalities of modern smartphones, such as communication, music streaming, gaming, and video playback [1]. Several publications in the Indian Journal of Otology suggest that mobile phone usage may contribute to sensorineural hearing loss [2].

According to the Indian Council of Medical Research, hearing impairment is rising in India, affecting one in every 12 individuals [3]. Many young adults today listen to portable listening devices at high volumes for extended periods, leading to prolonged exposure to loud noise. Sound levels exceeding 90 dB can sometimes cause hearing loss, with one of the critical consequences being difficulty understanding speech, which may result in anxiety and fatigue in social settings [4]. Studies have shown that individuals exposed to loud music for long durations experience a significant decline in cognitive function test scores [5].

The growing popularity of portable listening devices that connect directly to the ear is concerning, as it may significantly contribute to an increase in hearing loss among younger generations. A study by Portnuff et al. [6] found that the output levels of these devices can reach intensities that pose a substantial risk for noise-induced hearing loss. Portable music players have a detrimental effect on hearing thresholds, yet most students, despite being aware of the risks of loud noise exposure, tend to underestimate the impact of sound from MP3 players or mobile phones [7]. Since voluntary behavioral changes are challenging to implement, a strategic approach is necessary to prevent noise-induced hearing loss. Long-term exposure to loud music can also lead to additional auditory impairments such as ear infections, dizziness, and tinnitus [8].

Following the COVID-19 pandemic, there has been a notable increase in mobile phone and portable listening device usage among medical students. Many attend online lectures for a minimum of eight hours daily and subsequently use these devices to listen to music or podcasts for relaxation. As a result, prolonged exposure to loud noise has become routine, particularly as remote learning and work-from-home environments have become widespread. This habitual exposure may have detrimental effects on hearing, particularly among students, who are required to attend online classes for extended periods. Some studies suggest that this not only affects their hearing but also has negative implications for cognitive function and social interaction [9].

Given these concerns, this study aims to assess noise-induced hearing loss due to prolonged portable listening device usage, identify the earliest stages of hearing impairment, and examine gender-based differences in hearing loss among medical students in Puducherry. By conducting this research, we hope to raise awareness among younger generations about the harmful effects of prolonged exposure to high-volume sound on hearing health.

## Materials and methods

Study design

This cross-sectional study was conducted among medical students at Sri Venkateshwaraa Medical College Hospital and Research Centre, a tertiary care center in Puducherry, India. The study spanned four months, from January 2020 to April 2020.

Sampling

The patients were selected using the convenient sampling method. Considering a prevalence of hearing loss of 50% among medical students using listening devices [1], with an absolute margin of error of 5% and a 95% CI, the total sample size was calculated to be 236.

Inclusion criteria

All medical students aged 17-25 years who used listening devices were included in the study.

Exclusion criteria

Students who did not use portable listening devices, those with a history of ear disorders or chronic ototoxic drug intake, individuals with prior noise exposure, and those with a family history of hearing loss were excluded from the study. Additionally, students previously diagnosed with hearing loss were also excluded.

Data collection

After obtaining approval from the Institute Ethics Committee and informed written consent, construction workers who visited the hospital for conditions unrelated to the nose or ear were included in the study. The purpose of the study was explained to all participants, and informed written consent was obtained. A comprehensive clinical history of ear-related symptoms was recorded, followed by an otological examination. Students with ear diseases meeting the exclusion criteria were excluded from the study.

The study participants then underwent pure-tone audiometry (PTA) in a soundproof room in the Department of Otorhinolaryngology, using a Viola digital audiometer (Inventis, Padua, Italy). Air conduction thresholds were measured at frequencies of 125 Hz, 250 Hz, 500 Hz, 1 kHz, 2 kHz, 4 kHz, and 8 kHz, while bone conduction thresholds were assessed at 250 Hz, 500 Hz, 1 kHz, 2 kHz, and 4 kHz. The hearing thresholds were then compared, and the degree of hearing loss was evaluated.

Data analysis

Data entry was carried out using Epi-Data software, and statistical analysis was performed using IBM SPSS Statistics for Windows, Version 24.0 (Released 2017; IBM Corp., Armonk, NY, USA).

Ethical approval

The study protocol was approved by the Internal Human Ethics Committee of Sri Venkateshwaraa Medical College Hospital and Research Centre, Puducherry, India, under approval number 7/SVMCH/IEC/0820.

## Results

A total of 236 medical students participated in this study, ranging in age from 19 to 24 years, with a mean age of 21.1 ± 0.86 years. The majority of participants, 175 (74.1%), were in the 21-22 age group, followed by 43 (18.2%) in the 19-20 age group, and 18 (7.7%) in the 23-24 age group. Among the participants, 142 (60%) were female, while 94 (40%) were male, as shown in Table 1.

**Table 1:** Sociodemographic details of the study participants (n = 236)

Variable	Frequency (n)	Percentage (%)
Age (in years)		
19-20	43	18.2
21-22	175	74.1
23-24	18	7.7
Gender		
Male	142	60
Female	94	40

**Figure 1:**
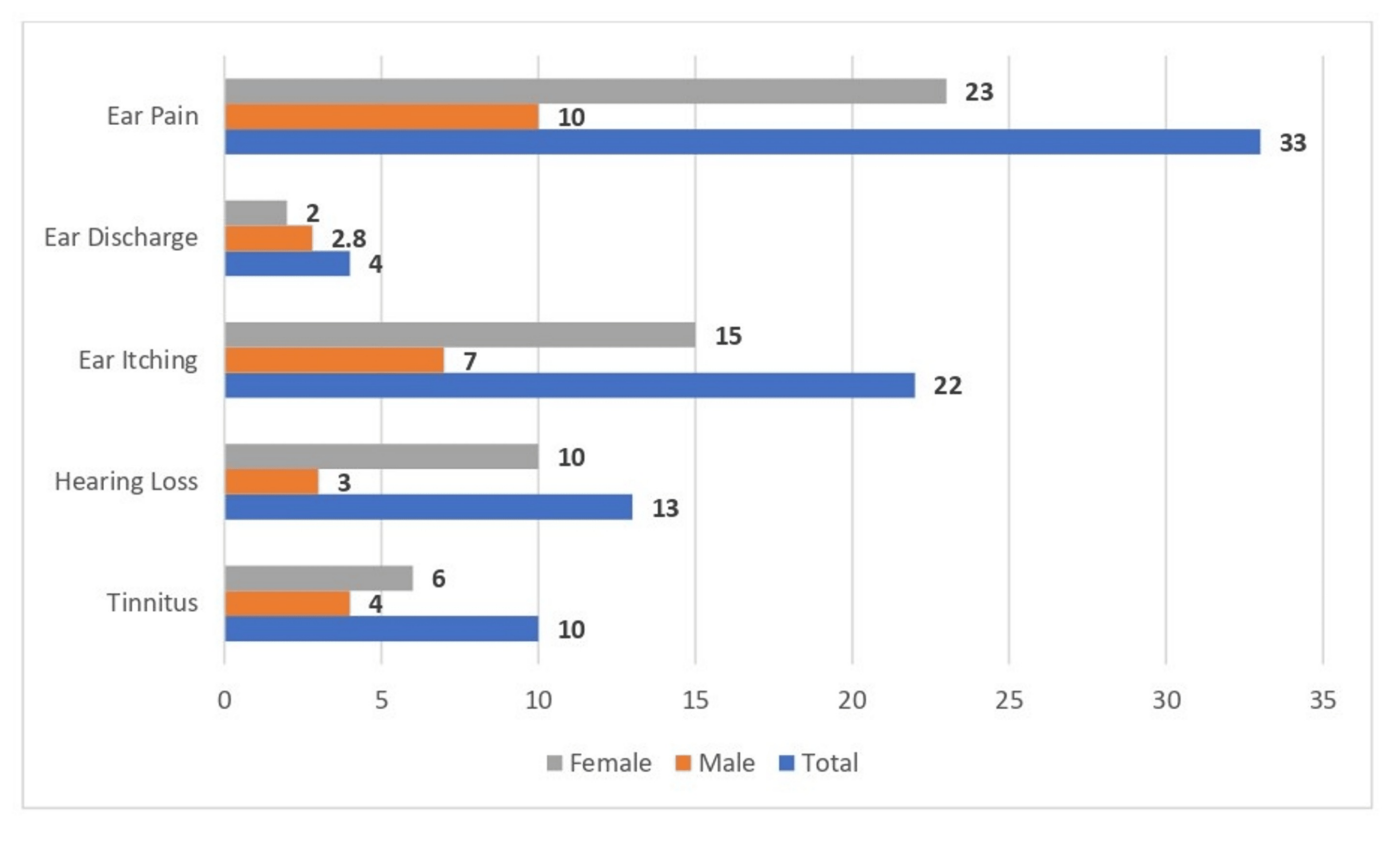
Distribution of study participants based on presenting symptoms

Approximately 33 (14%) of the study participants experienced ear pain, while 22 (9.3%) reported ear itching. Additionally, 13 (5.5%) experienced hearing loss, and 10 (4.2%) reported tinnitus while using portable listening devices. Only four (1.7%) participants had ear discharge, as shown in Figure 1.

The study found that the average duration of audio device use among participants was 3.66 ± 1.55 years. The majority of participants, 132 (56%), reported using portable listening devices for three to four years, while only 48 (20%) had been using them for less than two years. Notably, 87 (61%) of female participants and 45 (48%) of male participants had been using these devices for three to four years.

The average frequency of device usage per week was 5.33 ± 1.86 days. A significant proportion, 167 (71%), used the devices for more than four days per week, while only 26 (11%) used them for less than two days per week. Among them, 101 (71%) of females and 66 (70%) of males reported using portable listening devices more than four days per week.

The mean daily usage duration was 2.19 ± 1.23 hours. Most participants, 163 (69%), reported using the devices for less than two hours per day, with 107 (75%) of female participants falling into this category, as shown in Table 2.

**Table 2:** Frequency distribution of study participants based on the duration of personal audio device usage (n = 236)

Personal audio device variable	Total (%)	Male (%)	Female (%)
Duration (years)			
Mean ± SD	3.66 ± 1.55	3.94 ± 1.88	3.47 ± 1.26
≤2	48 (20)	18 (19)	30 (21)
3-4	132 (56)	45 (48)	87 (61)
>4	56 (24)	31 (33)	25 (18)
Days per week			
Mean ± SD	5.33 ± 1.86	5.19 ± 1.77	5.42 ± 1.92
≤2	26 (11)	9 (10)	17 (12)
3-4	43 (18)	19 (20)	24 (17)
>4	167 (71)	66 (70)	101 (71)
Hours per day			
Mean ± SD	2.19 ± 1.23	2.38 ± 1.31	2.06 ± 1.16
≤2	163 (69)	56 (60)	107 (75)
3-4	59 (25)	31 (33)	28 (20)
>4	14 (6)	07 (07)	07 (05)

Approximately 227 (96%) of the study participants had a PTA reading of >5 dB for bone conduction in the right ear, while 223 (94%) had a similar hearing loss in the left ear, with no significant sex-based differences. In the right ear, 139 (59%) of participants had hearing loss ranging between 15 and 20 dB, affecting 64 (68%) of males and 75 (53%) of females.

Only three (1%) participants exhibited hearing loss exceeding 20 dB for air conduction in the right ear. In the left ear, 97 (41%) had hearing loss within the 15-20 dB range, with a higher prevalence among males, as shown in Table 3.

**Table 3:** Frequency distribution of hearing loss in bone and air conduction among study participants based on PTA (n = 236) PTA, pure tone audiometry

PTA graph (dB)	Male, n (%)	Female, n (%)	Total, n (%)
	Bone conduction	Bone conduction	Bone conduction
Right			
≥5	90 (96)	137 (96)	227 (96)
<5	04 (04)	05 (04)	09 (04)
Left			
≥5	86 (91)	137 (96)	223 (94)
<5	08 (09)	05 (04)	13 (06)
	Air conduction	Air conduction	Air conduction
Right			
<15	29 (31)	65 (46)	94 (40)
15-20	64 (68)	75 (53)	139 (59)
>20	01 (01)	02 (01)	03 (01)
Left			
<15	45 (48)	89 (63)	134 (57)
15-20	47 (50)	50 (35)	97 (41)
>20	02 (02)	03 (02)	05 (02)

The study demonstrated a significant association between gender (p = 0.015), daily usage hours (p < 0.01), and air conduction thresholds among the study participants, as shown in Table 4.

**Table 4:** Association between demographic variables and PTA findings on hearing loss (n = 236) ^*^ p-value < 0.05 is considered statistically significant. PTA, pure tone audiometry

Puretone audiometry graph	Bone conduction			Air conduction		
	Average (dB)	F-value	p-value	Average (dB)	F-value	p-value
Age						
19-20	3.30 ± 0.81	0.693	0.187	14.37 ± 1.80	0.606	0.546
21-22	3.14 ± 1.02			14.14 ± 1.86		
23-24	3.02 ± 0.49			14.55 ± 1.49		
Gender						
Male	3.30 ± 1.08	3.002	0.07	14.56 ± 1.74	6.211	0.015^*^
Female	3.07 ± 0.86			13.97 ± 1.84		
Duration (in years)						
≤2	3.18 ± 0.88	0.051	0.95	14.45 ± 1.87	1.309	0.27
3-4	3.14 ± 0.99			14.02 ± 1.73		
>4	3.18 ± 0.93			14.36 ± 1.98		
Days per week						
≤2	2.84 ± 0.50	3.01	0.051	13.23 ± 1.24	5.651	0.07
3-4	3.00 ± 0.57			13.94 ± 1.29		
>4	3.26 ± 1.07			14.43 ± 1.96		
Hours per day						
≤2	3.08 ± 0.86	2.213	0.29	13.91 ± 1.54	11.734	<0.01^*^
3-4	3.31 ± 1.05			14.70 ± 2.27		
>4	3.50 ± 1.42			15.60 ± 1.77		

The study found a higher prevalence of hearing loss at 4,000 Hz and 8,000 Hz compared to other frequencies, suggesting a noise-induced etiology, as shown in Table 5.

**Table 5:** Association between the degree of hearing loss and various frequencies

Degree of hearing loss	Frequency (Hz)					
	250	500	1,000	2,000	4,000	8,000
<15	201	202	200	115	91	94
15-20	34	33	35	120	142	139
>20	1	1	1	1	3	3

## Discussion

Given the increasing amount of time people spend using earphones and mobile phones, as well as the potential adverse effects on health, investigating the impact of prolonged exposure to these devices is critically important. Mobile phone usage has not only become habitual but also essential in today’s challenging times.

This study included 236 participants, all aged between 17 and 24, with a history of earphone use. Among them, 74% were between the ages of 21 and 22, primarily because students formed the study’s main demographic. After carefully monitoring their audio device usage, it was observed that 14% of participants experienced ear pain, 9.3% reported ear itching, and 4.2% had tinnitus. Similarly, a study by Hutter et al. [10] found an increased risk of tinnitus with over four years of mobile phone exposure. In the present study, 4.2% of participants reported tinnitus. Additionally, Ueda et al. [11] examined symptoms in restaurant visitors exposed to sound frequencies of 20 kHz at 90-130 dB SPL, revealing high ratings for ear pain, discomfort, and irritation. These findings indicate that exposure to high-frequency sounds can lead to various auditory symptoms.

The average duration of audio device use among participants was recorded, with 56% using them for three to four years. This aligns with findings from Mourad Abd El-Mawgoud [12], where 42.68% of participants used portable listening devices for one to three years. Khullar et al. [13] also demonstrated an increased auditory brainstem response threshold among individuals who had used mobile phones for more than 30 minutes per day over the past decade. In this study, 71% of participants used their devices for more than four days per week, while only 11% used them for fewer than two days per week, consistent with findings from Das et al. [1]. Furthermore, 51% of students in Das et al.’s study used mobile phones for two or more hours daily, while 49% used them for less than two hours. Differences in sample size and exposure time may account for variations between the two studies. Another study by Basu et al. [14] found that 70% of participants used personal listening devices for less than one hour per day, while the remaining 30% used them for over two hours, results that are comparable to those of the present study.

Bone conduction values from PTA were measured, with 95% of participants showing values equal to or above 5 dB in both ears, while only 6% had values below 5 dB. Air conduction values from PTA were also recorded: in the right ear, nearly 60% had hearing loss between 15 and 20 dB, while in the left ear, 57% had values below 15 dB, and 41% fell within the 15-20 dB range. In a study by Das et al. [1], 74.4% of students were right-handed and primarily used their right ear for phone calls, while 25.6% predominantly used their left ear.

A significant association was found between gender (p = 0.015) and hours of daily device use (p < 0.01). In a study by Kim et al. [15], hearing thresholds of 462 Korean adolescents using portable music players were examined. While no gender differences were observed across all frequencies in the 12-19 age group, disparities were noted at 4 kHz. In the present study, a statistically significant difference was found between daily device usage and air conduction values (p < 0.05). Das et al. [1] analyzed 42 students who used mobile phones for at least two hours daily, showing that the mean PTA thresholds for air and bone conduction were higher in exposed ears compared to nonexposed ears - findings consistent with the current study. However, research by Das et al. [1] and Oktay and Dasdag [16] found no hearing threshold differences in individuals using mobile phones for 10-20 minutes per day but reported significant threshold increases in those using them for over two hours daily. Similarly, a study by Ramya et al. [17] observed a notable rise in hearing thresholds with prolonged mobile phone usage.

To mitigate noise-induced hearing loss, WHO recommends the “60-60 rule,” advising individuals to listen at no more than 60% volume for a maximum of 60 minutes per day.

Limitations

One limitation of this study is that it was conducted in a single study setting with a relatively small sample size, using a convenient sampling technique. A multilevel study design with a larger sample size and alternative sampling methods, such as stratified sampling, could have enhanced the study’s sensitivity and generalizability. Additionally, the study did not include a nonexposed control group for comparison, which could have provided a clearer understanding of the impact of prolonged mobile phone and earphone use on auditory health.

## Conclusions

A careful analysis of the study results revealed a statistically significant difference between the average daily use of personal devices and air conduction, as well as between air conduction and gender. These findings suggest that prolonged exposure to portable listening devices increases the risk of hearing impairment. Additionally, this study contributes to raising awareness about the dangers of extended exposure to loud music or noise, which can lead to hearing loss and other auditory issues.

To prevent noise-induced hearing loss, proactive measures should be taken to minimize prolonged exposure to these devices, ensuring they do not adversely impact the younger generation and future populations. The study also emphasizes the need for further research, particularly longitudinal studies with larger and more diverse sample sizes, to validate and expand upon these findings.
